# Supplementary material for: Solution structure, glycan specificity and of phenol oxidase inhibitory activity of Anopheles C-type lectins CTL4 and CTLMA2
Source: Sci Rep. 2019 Oct 23;9:15191. doi: 10.1038/s41598-019-51353-z (PMC6811590; doi:10.1038/s41598-019-51353-z)
Supplement: Supplementary file 1 — Supplementary information [file 41598_2019_51353_MOESM1_ESM.pdf]

Supplementary Information accompanying:

Solution structure, glycan specificity and of phenol oxidase inhibitory activity of *Anopheles* C-type lectins CTL4 and CTLMA2.

Ritika Bishnoi[1], Gregory L. Sousa[2], Alicia Contet[3,¶], Christopher J. Day[4], Chun-Feng David Hou[1], Lauren A. Profitt[5], Deepak Singla[6,†], Michael P. Jennings[4], Ann M. Valentine[5], Michael Povelones[2], Richard H. G. Baxter[1,3]*

Table S1: dsRNA and qPCR primers used

Name	Sequence
T7 Primers	
LacZ-T7 F	taatacgactcactatagggAGAATCCGACGGGTTGTTACT
LacZ-T7 R	taatacgactcactatagggCACCACGCTCATCGATAATTT
AgCTL4-T7 F	taatacgactcactatagggTGGTTTGATGCCGTGTCCT
AgCTL4-T7 R	taatacgactcactatagggAATAAATTGTCTCGGTTCATCATC
AgCTLMA2-T7 F	taatacgactcactatagggGCCTTTGCCCGTGCAAACCGTTC
AgCTLMA2-T7 R	taatacgactcactatagggTTGACAGATGAACGGTTTCTGCTG
AgTEP1-T7 F	taatacgactcactatagggTTTGTGGGCCTTAAAGCGCTG
AgTEP1-T7 R	taatacgactcactatagggACCACGTAACCGCTCGGTAAG
qPCR primers	
AgS7-qPCR F	GTGCGCGAGTTGGAGAAGA
AgS7-qPCR R	ATCGGTTTGGGCAGAATGC
AgCTL4-qPCR F	GCACGGGTACAGGGCTACTA
AgCTL4-qPCR R	GCGTGGTGTACAGCTTTCCT
AgCTLMA2-qPCR F	GCTGTCATCACAGTGGTTCG
AgCTLMA2-qPCR R	GGGTTTTGTTGAAGAATATCATCC
AgTEP1-qPCR F	AAAGCTGTTGCGTCAGGG
AgTEP1-qPCR R	TTCTCCCACACACCAAACGAA

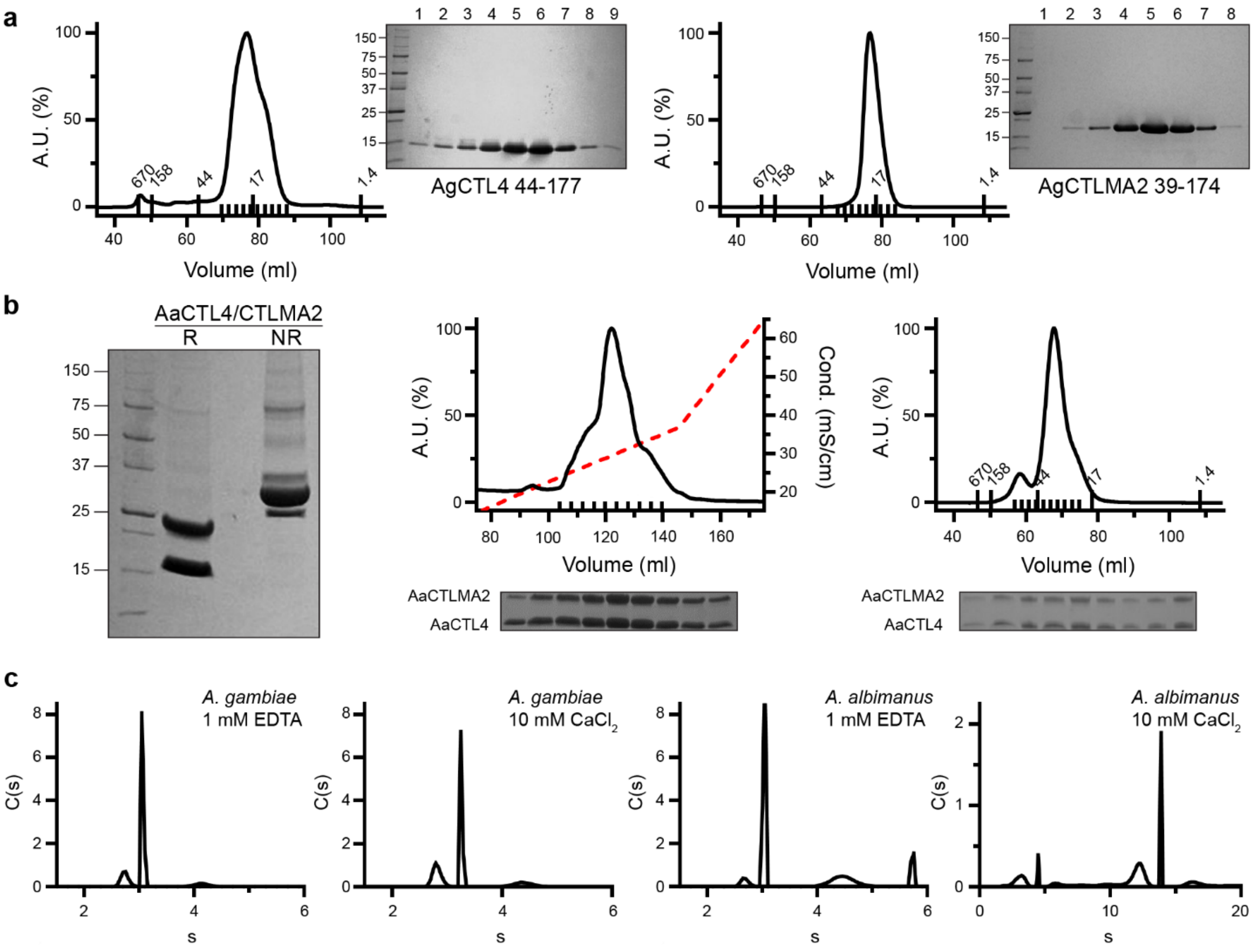

Figure S1. Purification of *An. gambiae* CTL4, CTLMA2 and *An. albimanus* CTL4/CTLMA2. (**b**) Reducing (R) and non-reducing (NR) SDS-PAGE, anion exchange (MonoQ 10/10) and size-exclusion (Superdex75 16/60) chromatogram for *An. albimanus* CTL4/CTLMA2. Small ticks indicate reducing SDS-PAGE peak fractions, large ticks indicate SEC MW standards. Representative of >5 independent experiments. (**b**) Plot of $C(s)$ vs. s for sedimentation velocity analytical ultracentrifugation of 0.5 mg/ml *An gambiae* and *An. albimanus* CTL4/CTLMA2, in 0.15 M NaCl, 20 mM Tris pH 7.5 and either 10 mM $CaCl_2$ (Ca-TBS) or 1 mM EDTA (EDTA-TBS). Results from a single experiment.
